# Mortality of patients infected with HIV in the intensive care unit (2005 through 2010): significant role of chronic hepatitis C and severe sepsis

**DOI:** 10.1186/s13054-014-0475-3

**Published:** 2014-08-27

**Authors:** José Medrano, Alejando Álvaro-Meca, Alexandre Boyer, María A Jiménez-Sousa, Salvador Resino

**Affiliations:** Departamento de Medicina, Universidad del País Vasco UPV/EHU, Vitoria-Gasteiz, Spain; Servicio de Urgencias, Hospital Universitario de Araba, Vitoria-Gasteiz, Spain; Unidad de Medicina Preventiva y Salud Pública, Facultad de Ciencias de la Salud, Universidad Rey Juan Carlos, Alcorcón, Madrid Spain; Université de Bordeaux, INSERM U657, Pharmaco-épidémiologie et évaluation de l’impact des produits de santé sur les populations, F-33000 Bordeaux cedex, France; Unidad de Infección Viral e Inmunidad, Centro Nacional de Microbiología, Instituto de Salud Carlos III, Majadahonda, Madrid Spain

## Abstract

**Introduction:**

The combination antiretroviral therapy (cART) has led to decreased opportunistic infections and hospital admissions in human immunodeficiency virus (HIV)-infected patients, but the intensive care unit (ICU) admission rate remains constant (or even increased in some instances) during the cART era. Hepatitis C virus (HCV) infection is associated with an increased risk for hospital admission and/or mortality (particularly those related to severe liver disease) compared with the general population. The aim of this study was to assess the mortality among HIV-infected patients in ICU, and to evaluate the impact of HIV/HCV coinfection and severe sepsis on ICU mortality.

**Methods:**

We carried out a retrospective study based on patients admitted to ICU who were recorded in the Minimum Basic Data Set (2005 through 2010) in Spain. HIV-infected patients (All-HIV-group (*n* = 1,891)) were divided into two groups: HIV-monoinfected patients (HIV group (*n* = 1,191)) and HIV/HCV-coinfected patients (HIV/HCV group (*n* = 700)). A control group (HIV(-)/HCV(-)) was also included (*n* = 7,496).

**Results:**

All-HIV group had higher frequencies of severe sepsis (57.7% versus 39.4%; *P* < 0.001) than did the control group. Overall, ICU mortality in patients with severe sepsis was much more frequent than that in patients without severe sepsis (other causes) at days 30 and 90 in HIV-infected patients and the control group (*P* < 0.001). Moreover, the all-HIV group in the presence or absence of severe sepsis had a higher percentage of death than did the control group at days 7 (*P* < 0.001), 30 (*P* < 0.001) and 90 (*P* < 0.001). Besides, the HIV/HCV group had a higher percentage of death, both in patients with severe sepsis and in patients without severe sepsis compared with the HIV group at days 7 (*P* < 0.001) and 30 (*P* < 0.001), whereas no differences were found at day 90. In a bayesian competing-risk model, the HIV/HCV group had a higher mortality risk (adjusted hazard ratio (aHR) = 1.44 (95% CI = 1.30 to 1.59) and aHR = 1.57 (95% CI = 1.38 to 1.78) for patients with and without severe sepsis, respectively).

**Conclusions:**

HIV infection was related to a higher frequency of severe sepsis and death among patients admitted to the ICU. Besides, HIV/HCV coinfection contributed to an increased risk of death in both the presence and the absence of severe sepsis.

**Electronic supplementary material:**

The online version of this article (doi:10.1186/s13054-014-0475-3) contains supplementary material, which is available to authorized users.

## Introduction

The introduction of combination antiretroviral therapy (cART) has dramatically decreased the morbidity/mortality associated with human immunodeficiency virus (HIV) infection in industrialized countries, leading to a higher prevalence of persons living with HIV [1]. Furthermore, cART leads to decreased opportunistic infections and hospital admissions [2,3]; but the intensive care unit (ICU) admission rate remains constant (or even increased in some instances) during the cART era [4-6]. This may be explained by the fact that HIV-infected patients live longer and are at higher risk of developing nonacquired immunodeficiency syndrome (AIDS) comorbid illnesses, as well as problems related to immune reconstitution inflammatory response syndrome and toxicities related to cART [4,7-9].

Moreover, the impact of HIV infection and cART on the ICU mortality remains controversial [10]. Thus, Chiang *et al.* [11] reported that outcome during the ICU stay was associated with CD4 count and sepsis, but was not associated with cART. However, Amâncio *et al.* [12] reported that the in-ICU mortality was significantly associated with cART in addition to comorbidities and septic shock. Besides, several authors have reported that HIV-related variables had scarce or no impact on death in patients admitted to the ICU, although they have been closely related to the long-term outcome [5,8,13,14]. Moreover, although Coquet *et al.* [15] reported an increased ICU survival in the cART era, Vincent *et al.* [14] reported unchanged overall ICU mortality, but significantly improved 3-month survival of HIV-infected patients admitted to the ICU in the cART era.

Hepatitis C virus (HCV) is an important cause of acute and chronic hepatitis worldwide. Unfortunately, one fourth of HCV-infected patients will progress to cirrhosis. HCV infection remains the leading cause of decompensated cirrhosis, hepatocellular carcinoma, and liver transplantation [16,17]. Chronic hepatitis C (CHC) is associated with an increased risk for hospital admission and/or mortality, particularly those related to severe liver disease [18,19]. This increased mortality rate still exists, even if HCV-specific treatment is administered [9]. Around 20% of HIV-infected individuals worldwide are chronically coinfected with HCV, with coinfection rates reaching as high as 90% in persons with a history of injection-drug use [20,21]. Because the course of HCV-associated liver disease may be accelerated in HIV/HCV-coinfected patients, HCV has emerged as a major cause of mortality in HIV/HCV-coinfected patients on cART [22]. Fortunately, new direct antiviral agents (DAAs) against HCV and the recognition of genetic factors that determine HCV clearance are opening a new era that has been compared with the cART era for HIV infection [23].

The aim of this study was to assess the mortality in HIV-infected patients admitted to the ICU during the modern cART era, and to evaluate the impact of HIV/HCV coinfection and severe sepsis on ICU mortality.

## Materials and methods

### Study design and data source

We carried out a retrospective cohort study of all consecutive HIV-infected patients older than 18 years who were admitted to the ICU in Spanish hospitals from January 1, 2005, to December 31, 2010.

We also selected a control group of HIV-uninfected patients admitted to the ICU in a proportion of 4:1 regarding to HIV-infected patients involved in the study. This method increases the statistical power and the accuracy of statistical tests [24-26] and is especially useful with a limited number of events. The control group (selective “cohort”) was obtained by random selection among the patients older than 18 years with negative results for HIV, HCV, and HBV testing. To avoid confounding factors, patients were matched for age, gender, trauma, and surgical conditions. More specifically, the selection of the control patients was performed by frequency [27], quartiles of age of HIV-infected patients, and assuming an approximate percentage of men and conditions influencing health status (surgical conditions and trauma).

Data were obtained from the records of the Minimum Basic Data Set (MBDS) of the National Surveillance System for Hospital Data in Spain, provided by the Spanish Ministry of Health. The MBDS is a clinical and administrative database containing information obtained and recorded at time of hospital discharge, with an estimated coverage of 97.7% and 25% of total hospital admissions to public and private hospitals, respectively [28]. The MBDS provides the encrypted patient identification number, sex, date of birth, dates of hospital admission and discharge, medical institutions providing the services, the diagnosis and procedure codes according to the *International Classification of Diseases, 9th ed, Clinical Modification* (ICD-9-CM), and outcome at discharge [29].

The data were treated with full confidentiality, according to Spanish legislation. MBDS is a nonpersonal data extract that is mandatory for hospitals to guarantee the epidemiologic knowledge necessary for driving national health system management. This database is regulated by an organic law that explains how institutions have to proceed with health-related personal data. In this setting, consent is not required because personal data are collected for the exercise of the functions proper to public administrations. The Spanish Ministry of Health confirmed that our study fulfilled all ethical considerations, according to Spanish legislation. The study was approved by the Research Ethics Committee (Comité de Ética de la Investigación y de Bienestar Animal) of the Instituto de Salud Carlos III (Madrid, Spain).

### ICD-9-CM codes and study groups

The ICD-9-CM codes that were used for defining the viral infection status were as follows: (i) HIV infection (042 or V08); (ii) HCV infection (ICD-9-CM codes 070.41, 070.44, 070.51, 070.54, 070.7x, or V02.62); and (iii) HBV infection (ICD-9-CM codes 070.2x, 070.3x, or V02.61).

We selected patients admitted to the ICU who were coded in the MBDS as MIV, which indicates Adult ICU. HBV infection was a criterion for exclusion. Next, we established several groups of patients according to their HIV and HCV status: (i) control group (randomly selected patients without HIV or HCV infections); (ii) all-HIV group (patients with or without HCV coinfection), which was divided into two groups: (a) HIV-monoinfected patients (HIV group) (patients exclusively infected with HIV (HCV infection was excluded)); (b) HIV/HCV-coinfected patients (HIV/HCV group) (patients exclusively coinfected with HIV and HCV).

### Outcome, follow-up, definitions

The primary outcome was ICU mortality. The secondary outcome was the presence of severe sepsis, which was defined by the presence of an infection-associated diagnosis and organ dysfunction, according to the criteria of Angus *et al*. [30], by using ICD-9-CM codes (see Additional file 1: Appendix 1 and 2, respectively. The MBDS provides the ICD-9-CM codes for Angus implementation, but not the date of diagnoses. Thus, we were unable to calculate the date of onset of severe sepsis, and severe sepsis was recorded all over the hospital stay.

Length of stay was obtained as the difference, in days, between date of hospital admission and date of discharge or death in the ICU. The day of hospital admission was considered day 0. Discharge on the same day was considered a 1-day stay. For patients admitted several times to the ICU, only the first admission (also called the index episode) was analyzed.

### Statistical analysis

Overall, results are presented as the median and interquartile range for continuous variables, and frequencies and percentages for categoric data. HIV-infected patients and HIV-uninfected patients were matched by frequencies, which allowed the use of statistical tests for independent groups. Categoric data and proportions were analyzed by using the χ2 test or Fisher Exact test, as required. A *t* test or Mann-Whitney *U* test was used to compare continuous variables. All tests were two-tailed with *P* values <0.05 considered significant.

The cumulative mortality rate at days 7, 30, and 90 in patients with ICU admission was calculated without considering censoring. This rate was estimated by dividing the number of deaths by the number of patients admitted to the ICU. Log-linear modeling for contingency tables was used to estimate main and interaction (moderator) effects independently. Moreover, we also calculated the probability of ICU death after taking censoring into account through a semiparametric bayesian model of competing risk [31], which was used to evaluate the association between HIV infection or HIV/HCV coinfection and the risk of ICU mortality, according to the presence of severe sepsis: (a) risk of ICU mortality with severe sepsis; and (b) risk of ICU mortality from other causes (excluding severe sepsis). Inference in our bayesian competing-risk model was based on Markov Chain Monte Carlo (MCMC) simulation algorithms [32].

Moreover, we used the following covariates for adjusting the model: (a) age, sex, trauma, surgical status, Charlson Co-morbidity Index (CCI) (see Additional file 1: Appendix 3), and number of organ failures and sites of infection (see Additional file 1: Appendix 4). This semiparametric bayesian model provides the survival probabilities and the hazard ratio (HR). When interpreting results on the basis of calculated survival probabilities, we took the presence of competing risks (severe sepsis and death) into account to prevent biased results [33].

All analyses were performed by using R statistical package version 3.0.2 (GNU General Public License; available at [34]) and BayesX software version 2.1 (GNU General Public License; available at [35]).

## Results

### Patient characteristics

Table 1 shows the epidemiologic and clinical characteristics of patients included in this study. The all-HIV group had a greater length of hospital stay than the control group (*P* < 0.001), as well as higher frequencies of alcohol/drug abuse, AIDS comorbidities (central nervous system disease, Kaposi sarcoma, non-Hodgkin lymphoma, and wasting syndrome) and CHC comorbidities (chronic liver disease and cirrhosis and decompensated cirrhosis) (*P* < 0.05) (Table 1a). Regarding HIV subgroups, the HIV/HCV group had higher frequencies of alcohol/drug abuse and CHC-related comorbidities than the HIV group (*P* < 0.05); whereas the HIV group had a longer hospital stay (*P* < 0.001) and a higher frequency of cancer (*P* < 0.05) than the HIV/HCV group (Table 1b).

**Table 1:** **Epidemiologic and clinical characteristics of patients admitted to the intensive care unit from 2005 to 2010**

	**1a: Non-HIV versus all HIV patients**			**1b: HIV versus HIV/HCV patients**		
	**Control group**	**All HIV group**	***P*** **value**	**HIV group**	**HIV/HCV group**	**p-value**
**Number of patients**	7,496	1,891		1,191 (63%)	700 (37%)	
**Gender (male)**	5,827 (77.73%)	1,466 (77.52%)	0.869	911 (76.49%)	555 (79.28%)	0.177
**Age (years)**	44 (36-49)	43 (38-48)	0.064	43 (38-50)	43 (39-47)	0.060
**Abuse of alcohol and drugs**	2,453 (32.72%)	942 (49.81%)	**<0.001**	494 (41.48%)	448 (64.00%)	**<0.001**
**Length of hospital stay (days)**	5 (1-25)	7 (2-21)	**<0.001**	9 (2-25)	5 (1-14)	**<0.001**
**Conditions influencing health status**						
Surgical conditions (V42, V45)	302 (4.02%)	80 (4.23%)	0.740	53 (4.45%)	27 (3.85%)	0.616
Trauma (E880* to E929*, E950 to E999*)	350 (4.66%)	97 (5.12%)	0.435	64 (5.37%)	33 (4.71%)	0.603
**CHC-related comorbidities**						
Chronic liver disease and cirrhosis	851 (11.35%)	248 (13.11%)	**0.036**	106 (8.90%)	203 (29.00%)	**<0.001**
Decompensated cirrhosis	736 (9.82%)	224 (11.85%)	**0.010**	82 (6.88%)	142 (20.29%)	**<0.001**
Liver cancer	16 (0.21%)	8 (0.42%)	0.174	2 (0.17%)	6 (0.86%)	0.062
Liver transplant	48 (0.64%)	11 (0.58%)	0.900	3 (0.25%)	8 (1.14%)	**0.031**
**AIDS-related comorbidities**						
Central nervous system disease	31 (0.41%)	24 (1.27%)	**<0.001**	15 (1.26%)	9 (1.29%)	1
Cancer	73 (0.97%)	91 (4.81%)	**<0.001**	72 (6.05%)	19 (2.71%)	**0.002**
Kaposi sarcoma	-	27 (1.43%)	**NA**	25 (2.10%)	2 (0.29%)	**0.003**
Non-Hodgkin lymphoma	73 (0.97%)	65 (3.44%)	**<0.001**	49 (4.11%)	16 (2.29%)	**0.048**
Wasting syndrome	151 (2.01%)	98 (5.18%)	**<0.001**	65 (5.46%)	33 (4.71%)	0.551

Values are expressed as absolute number (percentage) and median (percentile 25; percentile 75). Values were calculated by χ^2^ test and Mann-Whitney *U* test; and *P* values in bold indicate statistically significant differences between groups. HCV*,* hepatitis C virus; HIV, human immunodeficiency virus.

Table 2 shows the prevalence of processes related to severe sepsis and opportunistic infections among patients admitted to the ICU in this study. The all-HIV group had a higher frequency of severe sepsis (*P* < 0.001), acute organ dysfunctions (except for neurologic dysfunction) (*P* < 0.05), respiratory system infections (*P* < 0.001), central nervous system infections (*P* < 0.001), and opportunistic infections (*P* < 0.001) than the control group (Table 2a). Moreover, the HIV/HCV group had a lower frequency of severe sepsis (*P* = 0.028), respiratory system infections (*P* < 0.001), genitourinary tract infections (*P* = 0.016), and opportunistic infections (*P* < 0.001); and a higher frequency of neurologic and hepatic dysfunction (*P* < 0.05), and digestive system infections (*P* < 0.001) than the HIV group (Table 2b).

**Table 2:** **Summary of diagnoses related to severe sepsis and opportunistic infections in the intensive care unit from 2005 to 2010**

	**2a: Non-HIV versus all HIV patients**			**2b: HIV versus HIV/HCV patients**		
	**Control group**	**All-HIV group**	***P*** **value**	**HIV group**	**HIV/HCV group**	***P*** **value**
**Severe sepsis**	2,954 (39.41%)	1,092 (57.75%)	**<0.001**	711 (59.70%)	381 (54.43%)	**0.028**
**Acute organ dysfunction**						
Respiratory	4,882 (65.13%)	1,442 (76.26%)	**<0.001**	917 (76.99%)	525 (75.00%)	0.353
Cardiovascular	2,463 (32.85%)	811 (42.88%)	**<0.001**	498 (41.81%)	313 (44.71%)	0.237
Renal	2,211 (29.49%)	608 (32.15%)	**0.026**	374 (31.40%)	234 (33.42%)	0.389
Hematologic	937 (12.50%)	326 (17.23%)	**<0.001**	208 (17.46%)	118 (16.85%)	0.783
Metabolic	693 (9.24%)	221 (11.68%)	**0.001**	139 (11.67%)	82 (11.71%)	1
Neurologic	793 (10.57%)	216 (11.42%)	0.309	119 (9.99%)	97 (13.85%)	**0.013**
Hepatic	731 (9.75%)	214 (11.31%)	**0.047**	117 (9.82%)	97 (13.85%)	**0.009**
**Site of infection**						
Respiratory	1,775 (23.67%)	900 (47.59%)	**<0.001**	620 (52.05%)	280 (40.00%)	**<0.001**
Digestive	950 (12.67%)	219 (11.58%)	0.281	102 (8.56%)	117 (16.71%)	**<0.001**
Genitourinary	275 (3.66%)	80 (4.23%)	1	61 (5.12%)	19 (2.71%)	**0.016**
Central nervous system	75 (1.01%)	79 (4.17%)	**<0.001**	50 (4.19%)	29 (4.14%)	1
Skin, soft tissue, or bone	153 (2.04%)	32 (1.69%)	0.377	19 (1.59%)	13 (1.85%)	0.809
Circulatory	77 (1.02%)	17 (0.89%)	0.710	13 (1.09%)	4 (0.57%)	0.365
**Opportunistic infections**	1,428 (19.05%)	931 (49.23%)	**<0.001**	645 (54.16%)	286 (40.86%)	**<0.001**
Candidiasis (pulmonary or esophageal)	78 (1.02%)	82 (4.34%)	**<0.001**	59 (4.95%)	23 (3.29%)	0.109
Cryptococcosis (extrapulmonary)	1 (0.01%)	27 (1.43%)	**<0.001**	18 (1.51%)	9 (1.29%)	0.843
Cytomegalovirus (other than liver, spleen, or nodes)	40 (0.53%)	80 (4.23%)	**<0.001**	70 (5.88%)	10 (1.43%)	**<0.001**
*Mycobacterium tuberculosis*	76 (1.01%)	82 (4.34%)	**<0.001**	60 (5.04%)	22 (3.14%)	0.066
*Pneumocystis jirovecii pneumonia*	12 (0.16%)	225 (11.90%)	**<0.001**	195 (16.37%)	30 (4.29%)	**<0.001**
Pneumonia (bacterial), recurrent	1,336 (17.82%)	579 (30.62%)	**<0.001**	367 (30.81%)	212 (30.29%)	0.850
Toxoplasmosis of brain	3 (0.04%)	40 (2.12%)	**<0.001**	33 (2.77%)	7 (1.00%)	**0.016**

Values are expressed as absolute number (percentage). *P* values were calculated by χ^2^ test, and *P* values in bold indicates statistically significant differences between groups. HCV, hepatitis C virus; HIV, human immunodeficiency virus.

### Mortality among patients admitted to the ICU

Table 3 shows the cumulative mortality rate at days 7, 30, and 90 in patients admitted to the ICU. Overall, the ICU mortality in patients with severe sepsis was much more frequent than in patients without severe sepsis (other causes) at days 30 and 90 for both the all-HIV group and the control group (*P* < 0.001; Table 3a), and the HIV group and the HIV/HCV group (*P* < 0.001; Table 3b).

**Table 3:** **Summary of the cumulative mortality rate at days 7, 30, and 90 in patients admitted to the ICU from 2005 to 2010 according to the presence of severe sepsis**

		**3a: Non-HIV versus all-HIV patients**			**3b: HIV versus HIV/HCV patients**		
		**Control group**	**All HIV group**	***P*** **value** ^**(a)**^	**HIV group**	**HIV/HCV group**	***P*** **value** ^**(b)**^
		**(No. = 7,496)**	**(No. = 1,891)**		**(No. = 1.191)**	**(No. = 700)**	
**Day 7**	**Number of deaths** ^(*)^	2,162	690		363	327	
	**Cumulative mortality rate** ^(**)^						
	**All patients**	28.8 (27.6; 30.1)	36.5 (33.8; 39.2)	**<0.001**	30.5 (27.3; 33.6)	46.7 (41.6; 51.8)	**<0.001**
	**Patients with severe sepsis**	26.1 (24.3; 27.9)	35.3 (31.7; 38.7)	**<0.001**	29.2 (25.4; 33.2)	46.5 (39.6; 53.3)	**<0.001**
	**Patients without severe sepsis**	30.6 (28.9; 32.2)	38.2 (33.9; 42.5)	**<0.001**	32.3 (27.2; 37.4)	47.1 (39.5; 54.5)	**<0.001**
	***P*** **value** ^**(c)**^	**<0.001**	0.210		0.292	0.941	
**Day 30**	**Number of deaths** ^(*)^	3819	1285		765	520	
	**Cumulative mortality rate** ^(**)^						
	**All patients**	50.9 (49.3; 52.5)	67.9 (64.2; 71.7)	**<0.001**	64.2 (59.7; 68.8)	74.3 (67.9; 80.7)	**<0.001**
	**Patients with severe sepsis**	64.0 (61.1; 66.9)	73.8 (68.7; 78.9)	**<0.001**	69.5 (63.3; 75.6)	81.9 (72.8; 90.9)	**<0.001**
	**Patients without severe sepsis**	42.4 (40.5; 44.3)	59.9 (54.6; 65.3)	**<0.001**	56.4 (49.7; 63.2)	65.2 (56.3; 74.1)	**0.016**
	***P*** **value** ^**(c)**^	**<0.001**	**<0.001**		**<0.001**	**<0.001**	
**Day 90**	**Number of deaths** ^(*)^	4419	1531		958	573	
	**Cumulative mortality rate** ^(**)^						
	**All patients**	58.9 (57.2; 60.7)	80.9 (76.9; 85.1)	**<0.001**	80.4 (75.3; 85.5)	81.9 (75.1; 88.6)	0.484
	**Patients with severe sepsis**	80.6 (77.3; 83.8)	90.7 (85.1; 96.4)	**<0.001**	90.7 (83.7; 97.7)	90.8 (81.2; 99.9)	0.999
	**Patients without severe sepsis**	44.9 (42.9; 46.8)	67.6 (61.8; 73.3)	**<0.001**	65.2 (57.9; 72.4)	71.2 (61.9; 80.4)	0.092
	***P*** **value** ^**(c)**^	**<0.001**	**<0.001**		**<0.001**	**<0.001**	

Values were expressed as follow: ^(*)^absolute count; ^(**)^percentage and 95% confidence interval (95% CI).

*P*-values were calculated by χ^2^ test: ^(a)^differences between control group and all HIV group; ^(b)^differences between HIV-group and HIV/HCV group; ^(c)^differences between severe sepsis and other causes. *P*-values in bold indicates statistically significant differences between groups. HCV*,* hepatitis C virus; HIV*,* human immunodeficiency virus.

Moreover, the all-HIV group had a higher percentage of death in all analyzed groups: all patients, patients with severe sepsis, and patients without severe sepsis (other causes) at days 7 (*P* < 0.001), 30 (*P* < 0.001), and 90 (*P* < 0.001) in comparison to the control group (Table 3a). Besides, the HIV/HCV group had a higher percentage of death for all patients, patients with severe sepsis, and patients without severe sepsis (other causes) than the HIV group at days 7 (*P* < 0.001) and 30 (*P* < 0.001), whereas no differences were found at day 90 (Table 3b).

The possible interaction between the virologic status (HIV^-^, HIV^+^, and HIV^+^/HCV^+^) and severe sepsis (present and absent) for death at 7, 30, and 90 days was evaluated. However, we did not find any significant interaction (data not shown), analyzing the main effects independently.

### Risk of death in the ICU among HIV-infected patients

**Figure 1:**
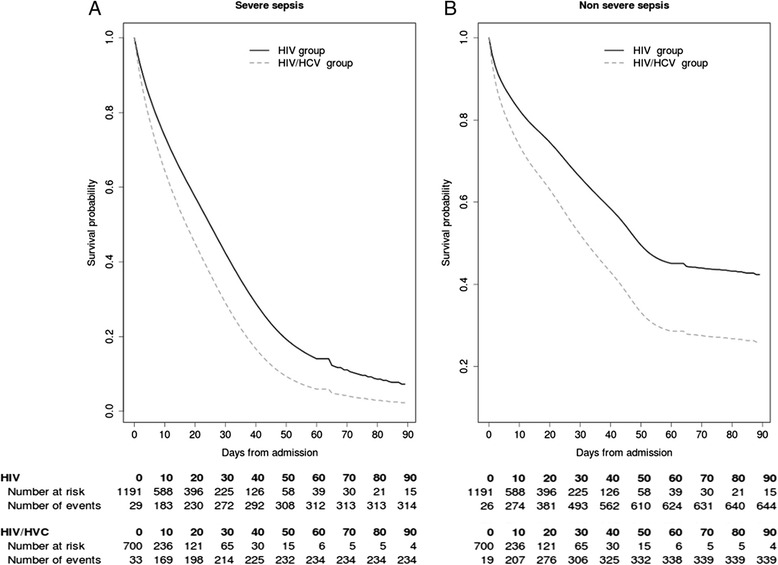
**Estimated survival for HIV-infected patients who were admitted to the intensive care unit (ICU) from 2005 to 2010 in Spain, stratified by the presence of severe sepsis (A) or absence of severe sepsis (B).** Survival functions were calculated with a competing-risk model that was developed with a semiparametric bayesian model. HCV, hepatitis C virus; HIV, human immunodeficiency virus.

Figure 1 shows the estimated survival function for patients admitted to the ICU, stratified by HIV status (HIV/HCV group versus HIV group), in the presence of competing risks: (i) ICU mortality with severe sepsis (Figure 1A), and (ii) ICU mortality from other causes (without severe sepsis) (Figure 1B). The estimated survival was lower in the HIV/HCV group than in the HIV group, regardless of the type of competing risk considered. Thus, the HIV/HCV group had higher mortality risk than the HIV group, both in presence of severe sepsis (adjusted hazard ratio (aHR) = 1.44 (95% CI = 1.30 to 1.59)) and in the absence of severe sepsis (other causes) (aHR = 1.57 (95% CI = 1.38 to 1.78)).

## Discussion

In this study, the major findings were as follows: (a) HIV-infected patients (all-HIV group) had higher frequencies of severe sepsis and opportunistic infections than did HIV-seronegative patients (control group); (b) HIV-infected patients had higher mortality than did the control group, independent of the presence of severe sepsis; (c) HIV/HCV coinfection (about one third of HIV-infected patients) had increased risk of ICU mortality, regardless of the presence or absence of severe sepsis.

The spectrum of critical illness in HIV-infected patients has changed since the introduction of cART [2,3]. At the beginning of the HIV epidemic, patients admitted to the ICU were mostly young individuals with advanced AIDS-related diseases; but in the cART era, the etiology of ICU admissions changed, whereby fewer patients were admitted to the ICU as a result of opportunistic infections [8,14]. A continual shift occurred toward ICU admissions for non-AIDS-related diagnoses [4]. Among them, sepsis has been responsible for about 12% to 31% of HIV-infected patient admissions to the ICU, and is associated with a worse prognosis [11,36,37]. In our study, we found that HIV-infected patients had a higher frequency of opportunistic infections than did patients in the control group; despite being in the late period of cART. Additionally, HIV-infected patients had higher frequency of severe sepsis, worse prognosis, and a higher death rate than HIV-seronegative subjects.

ICU management of HIV-infected patients in the pre-cART era was widely perceived as futile, as ICU mortality was about 70% [38]. Subsequently, cART did not seem to improve survival in the ICU compared with the pre-cART era [14,39]. In our study, HIV-infected patients had higher ICU mortality than the control group. These differences were regardless of the presence of severe sepsis, although it is noted that the ICU mortality was much more frequent in patients with severe sepsis than in patients without severe sepsis (other causes). These data are consistent with several recent reports [12,40,41]. Akgun *et al.* [40] found higher frequency of medical ICU admission, 30-day mortality, and mechanical ventilation in HIV-infected compared with HIV-uninfected patients [40]. Furthermore, Silva *et al.* [41] described a more severe course of sepsis as well as a higher ICU mortality in HIV-infected patients, and Amancio *et al.* [12]. reported that septic shock was associated with higher ICU mortality. Therefore, according to our data and previously published reports, it is not clear whether cART is associated with an improved survival. In our study, we had no information about compliance with cART, and we could not distinguish between patients with lack of cART initiation and patients with lack of cART benefit.

In our study, when severe sepsis and mortality in the HIV-group were compared with the HIV/HCV group, the data were slightly confusing. The frequency of severe sepsis in the HIV-group and the HIV/HCV group were both higher than 50%, and around 5% higher for the HIV group. Conversely, the ICU mortality was higher in the HIV/HCV group than in the HIV group at days 7 and 30, but no differences were found at day 90. We have tried to clarify these complex results through a competing-risk model, in which we operated with two different outcome variables (severe sepsis and death) that compete among themselves to prevent biased results (see Statistical Analysis section). This analysis shows that HIV/HCV coinfection (HIV/HCV group) had an increased risk of death compared with HIV-monoinfected patients (HIV group), regardless of the presence of severe sepsis. Thus, the HIV/HCV status *per se* seems to be deleterious, independent of severe sepsis.

Liver disease has become a major cause of morbidity and mortality among HIV/HCV-coinfected patients on cART, because HIV infection tends to modify the natural history of CHC toward a faster progression of liver fibrosis than for HCV-monoinfected patients [22,42,43]. In our study, about 50% of HIV/HCV-coinfected patients had comorbidities related to CHC and end-stage liver disease (ESLD), possibly because of the progressive deterioration from the underlying chronic liver disorder. Hepatic cirrhosis has been reported to be a major independent predictor of ICU mortality, and liver dysfunction results in a greater mortality burden than HIV status [15]. Furthermore, evidence suggests that during cirrhosis, sepsis is accompanied by a markedly imbalanced cytokine response, which converts responses that are normally beneficial for fighting infections into excessive, damaging inflammation [44]. Also, patients with ESLD have enhanced intestinal permeability (leading to translocation of bacteria and their products), imbalanced immune reaction, and aggravated intrahepatic microcirculatory dysfunction and hyperdynamic state, which cause toxin accumulation and immune dysfunction that might further enhance and perpetuate end-stage organ dysfunction [45]. Nevertheless, the occurrence of life-threatening conditions in cirrhosis patients is frequent and would explain higher mortality rates in the HIV/HCV group. [46]. Then, CHC and cirrhosis should be considered targets for improving the survival of HIV-infected patients in the ICU in the future.

The prognostic factors of mortality in HIV-infected patients admitted to the ICU are acute illness severity, poor functional status, low albumin rate, and respiratory failure requiring mechanical ventilation [47-53]. However, specific HIV characteristics (CD4 cell count, plasma HIV-RNA load, HIV-related diagnosis, or antiretroviral therapy) have not been clearly identified as predictors of ICU mortality [4,15,37,38,49-52,54-59], although some reports have associated CD4^+^ cell count with mortality risk [11,37]. Our study was retrospective, and the acquisition of the clinical data related to HIV infection and UCI was unavailable from MBDS records. In addition, other variables are difficult to control, such as the HCV infection, which is strongly linked to intravenous drug use, and the differences between HIV monoinfection and HIV/HCV coinfection could partially reflect drug use.

In our study, the time to death or discharge was calculated from hospital rather than ICU admission because the date of ICU admission was not recorded in the MBDS. An immortal time bias could have occurred [60,61], because some patients may have been admitted directly to ICU, whereas other patients may have survived between hospital and ICU admission, and this time period was included in the observation time. However, this theoretic bias should be well balanced in all groups, and only affect the basic value of survival time.

Another limitation due to the use of administrative databases is the inaccuracy in differentiating etiologies of diseases and the reporting of organ dysfunction, engendering confusion bias. In this context, grouping of ICD-9-CM codes into comorbidities, organ dysfunction, and site of infection (Additional file 1: Appendices 1 through 6) may have represented the best approach to solve this issue. However, the MBDS also provides certain advantages by being a national clinical administrative database, which represents large populations from developed countries. This database has already proven its usefulness in previous assessments of outcomes among patients admitted to the ICU [62,63]. Additionally, it allows the detection of trends in important public health issues.

## Conclusions

In conclusion, HIV infection was related to higher frequency of severe sepsis and death among patients admitted to the ICU. Also, HIV/HCV coinfection contributed to an increased risk of death in both the presence and absence of severe sepsis.

## Key messages

1. HIV-infected patients had higher frequencies of severe sepsis and opportunistic infections than did HIV-seronegative patients.
2. HIV-infected patients had higher mortality than the control group, independent of the presence of severe sepsis.
3. HIV/HCV coinfection (about one third of HIV-infected patients) had increased risk of ICU mortality, regardless of the presence of severe sepsis.

## Additional file

Additional file 1:
**Appendix 1.** *International Classification of Diseases, 9th Revision, Clinical Modification* (ICD-9-CM) codes for bacterial and fungal infections. **Appendix 2.** *International Classification of Diseases, 9th Revision, Clinical Modification* (ICD-9-CM) codes for acute organ dysfunction. **Appendix 3.** *International Classification of Diseases, 9th Revision, Clinical Modification* (ICD-9-CM) coding algorithms for Charlson comorbidities. **Appendix 4.** *International Classification of Diseases, 9th Revision, Clinical Modification* (ICD-9-CM) codes were used to identify the source of infection causing sepsis. **Appendix 5.** *International Classification of Diseases, 9th Revision, Clinical Modification* (ICD-9-CM) codes for AIDS and CHC related diagnoses. **Appendix 6.** *International Classification of Diseases, 9th Revision, Clinical Modification* (ICD-9-CM) codes for comorbid diseases.

## Abbreviations

AIDS: Acquired immunodeficiency syndrome
cART: combination antiretroviral therapy
CCI: Charlson co-morbidity index
CHC: chronic hepatitis C
DAA: direct antiviral agent
ESLD: end-stage liver disease
HCV: hepatitis C virus
HIV: human immunodeficiency virus
ICD-9-CM: International Classification of Diseases, 9th ed, Clinical Modification
ICU: intensive care unit
MBDS: minimum basic data set
