# Supplementary material for: Mortality of patients infected with HIV in the intensive care unit (2005 through 2010): significant role of chronic hepatitis C and severe sepsis
Source: Crit Care. 2014 Aug 27;18(4):475. doi: 10.1186/s13054-014-0475-3 (PMC4176576; doi:10.1186/s13054-014-0475-3)
Supplement: Additional file 1: — Appendix 1. International Classification of Diseases, 9th Revision, Clinical Modification (ICD-9-CM) codes for bacterial and fungal infections. Appendix 2. International Classification of Diseases, 9th Revision, Clinical Modification (ICD-9-CM) codes for acute organ dysfunction. Appendix 3. International Classification of Diseases, 9th Revision, Clinical Modification (ICD-9-CM) coding algorithms for Charlson comorbidities. Appendix 4. International Classification of Diseases, 9th Revision, Clinical Modification (ICD-9-CM) codes were used to identify the source of infection causing sepsis. Appendix 5. International Classification of Diseases, 9th Revision, Clinical Modification (ICD-9-CM) codes for AIDS and CHC related diagnoses. Appendix 6. International Classification of Diseases, 9th Revision, Clinical Modification (ICD-9-CM) codes for comorbid diseases. [file 13054_2014_475_MOESM1_ESM.pdf]

Supplementary Digital Content 1. Appendix 1-5

Appendix 1. *International Classification of Diseases, 9th Revision, Clinical Modification* (ICD-9-CM) codes for bacterial and fungal infections. Adapted from Angus et al. [1].

ICD-9-CM Code	ICD-9-CM Code Description
001	Cholera
002	Typhoid/paratyphoid fever
003	Other salmonella infection
004	Shigellosis
005	Other food poisoning
008	Intestinal infections due to other organisms
009	Ill-defined intestinal infection
013	Central nervous system tuberculosis
014	Tuberculosis of intestines peritoneum and mesenteric glands
015	Tuberculosis of bones and joints
016	Tuberculosis of genitourinary system
017	Tuberculosis of other organs
018	Miliary tuberculosis
020	Plague
021	Tularemia
022	Anthrax
023	Brucellosis
024	Glanders
025	Melioidosis
026	Rat-bite fever
027	Other bacterial zoonoses
030	Leprosy
031	Diseases due to other mycobacteria
032	Diphtheria
033	Whooping cough
034	Streptococcal throat/scarlet fever
035	Erysipelas
036	Meningococcal infection
037	Tetanus
038	Septicemia
039	Actinomycotic infections
040	Other bacterial diseases
041	Bacterial infection in other diseases not specified
090	Congenital syphilis
091	Early syphilis symptomatic
092	Early syphilis latent
093	Cardiovascular syphilis
094	Neurosyphilis
095	Other forms of late syphilis with symptoms
096	Late syphilis, latent
097	Other and unspecified syphilis
098	Gonococcal infections
100	Leptospirosis
101	Vincent’s angina

102	Yaws
103	Pinta
104	Other spirochetal infection
110	Dermatophytosis
111	Dermatomycosis other and unspecified
112	Candidiasis
114	Coccidioidomycosis
115	Histoplasmosis
116	Blastomycotic infection
117	Other mycoses
118	Opportunistic mycoses
320	Bacterial meningitis
321	Meningitis due to other organisms
324	Central nervous system abcess
325	Phlebitis of intracranial sinus
360.0	Purulent endophthalmitis
376.0	Acute inflammation of orbit
380.14	Malignant otitis externa
383.0	Acute mastoiditis
420	Acute pericarditis
421	Acute or subacute endocarditis
451	Phlebitis and thrombophlebitis
461	Acute sinusitis
462	Acute pharyngitis
463	Acute tonsillitis
464	Acute laryngitis/tracheitis
465	Acute upper respiratory infection of multiple sites/not otherwise specified
475	Peritonsillar abscess
481	Pneumococcal pneumonia
482	Other bacterial pneumonia
485	Bronchopneumonia with organism not otherwise specified
486	Pneumonia, organism not otherwise specified
491.21	Obstructive chronic bronchitis with (acute) exacerbation
491.22	Obstructive chronic bronchitis with acute bronchitis
494	Bronchiectasis
510	Empyema
513	Abscess of lung and mediastinum
522.5	Periapical abscess without sinus
522.7	Periapical abscess with sinus
526.4	Inflammatory conditions of the jaw
527.3	Abscess of the salivary glands
528.3	Cellulitis and abscess of oral soft tissue
540	Acute appendicitis
541	Appendicitis not otherwise specified
542	Other appendicitis
562.01	Diverticulitis of the small intestine without hemorrhage
562.03	Diverticulitis of the small intestine with hemorrhage
562.11	Diverticulitis of colon without hemorrhage
562.13	Diverticulitis of colon with hemorrhage
566	Abscess of the anal and rectal regions

567	Peritonitis
569.5	Intestinal abscess
569.61	Infection of colostomy or enterostomy
569.83	Perforation of intestine
572.0	Abscess of liver
572.1	Portal pyemia
575.0	Acute cholecystitis
590	Kidney infection
599.0	Urinary tract infection not otherwise specified
601	Prostatic inflammation
604	Orchitis and epididymitis
614	Female pelvic inflammation disease
615	Inflammatory diseases of uterus except cervix
616	Inflammatory disease of cervix vagina and vulva
616.3	Abcess of Bartholin's gland
616.4	Other abcess of vulva
634.0	Spontaneous abortion, complicated by genital tract and pelvic infection
635.0	Legally induced abortion, complicated by genital tract and pevic infection
636.0	Illegally induced abortion, complicated by genital tract and pelvic infection
637.0	Unspecified abortion, complicated by genital tract and pelvic infection
638.0	Failed attempted abortion, complicated by genital tract and pelvic infection
639.0	Complications following abortion and ectopic and molar pregnancies,genital and pelvic infection
646.6	Infections of genitourinary tract in pregnancy
658.4	Infection of amniotic cavity
670	Major puerperal infection
675.1	Abscess of breast
681	Cellulitis, finger/toe
682	Other cellulitis or abscess
683	Acute lymphadenitis
685.0	Pilonidal cyst, with abscess
686	Other local skin infection
711.0	Pyogenic arthritis
728.86	Necrotizing fasciitis
730	Osteomyelitis
790.7	Bacteremia
958.3	Posttraumatic wound infection, not elsewhere classified
996.6	Infection or inflammation of device/graft
998.5	Postoperative infection
999.3	Infectious complication of medical care not otherwise classified

Appendix 2. *International Classification of Diseases, 9th Revision, Clinical Modification* (ICD-9-CM) codes for acute organ dysfunction. Adapted from Angus et al. [1].

Organ System	ICD-9-CM Code	ICD-9-CM Code Description
Cardiovascular	427.5	Cardiac arrest
	458.0	Orthostatic hypotension
	458.8	Other specified hypotension
	458.9	Hypotension, unspecified
	785.5	Shock without mention of trauma
	796.3	Hypotension, transient
Hematologic	286.2	Disseminated intravascular coagulation
	286.6	Defibrination syndrome
	286.9	Other and unspecified coagulation defects
	287.4	Secondary thrombocytopenia
	287.5	Thrombocytopenia, unspecified
	790.92	Abnormal coagulation profile
Hepatic	570	Acute and subacute necrosis of liver
	572.2	Hepatic encephalopathy
	573.3	Hepatitis (septic & not elsewhere classified)
	573.4	Hepatic infarction
Neurologic	293	Transient organic psychosis
	348.1	Anoxic brain damage
	348.3	Encephalopathy, acute
	780.01	Coma
	780.09	Altered consciousness, unspecified
	89.14	Electroencephalography
Renal	580.x	Acute glomerulonephritis
	584.x	Acute renal failure
	586	Renal shutdown, renal failure unspecified
	39.95	Hemodialysis
Respiratory	518.5	Pulmonary insufficiency following trauma and surgery
	518.8	Respiratory failure
	786.03	Apnea
	799.1	Respiratory arrest
	786.09	Respiratory insufficiency
	96.7	Other Continuous Invasive Mechanical Ventilation
	96.04	Endotracheal intubation (emergency procedure)
	93.90	Continuous positive airway pressure°
Metabolic	276.2	Acidosis, metabolic or lactic

ICD-9-CM, International Classification of Diseases, Ninth Revision, Clinical Modification.

Appendix 3. . *International Classification of Diseases, 9th Revision, Clinical Modification* (ICD-9-CM) coding algorithms for Charlson comorbidities. Adapted from Quan et al. [2].

Comorbidities	ICD-9-CM
Myocardial infarction	410.x, 412.x
Congestive heart failure	428.x
Peripheral vascular disease	443.9, 441.x, 785.4, V43.4, Procedure 38.48
Cerebrovascular disease	430.x–438.x
Dementia	290.x
Chronic pulmonary disease	490.x–505.x, 506.4
Rheumatic disease	710.0, 710.1, 710.4, 714.0–714.2, 714.81, 725.x
Peptic ulcer disease	531.x–534.x
Mild liver disease	571.2, 571.4–571.6
Diabetes without chronic complication	250.0–250.3, 250.7
Diabetes with chronic complication	250.4–250.6
Hemiplegia or paraplegia	344.1, 342.x
Renal disease	582.x, 583–583.7, 585.x, 586.x, 588.x
Any malignancy, including lymphoma and leukemia, except malignant neoplasm of skin	140.x–172.x, 174.x.–195.8, 200.x–208.x
Moderate or severe liver disease	456.0–456.21, 572.2–572.8
Metastatic solid tumor	196.x–199.1
AIDS/HIV	042.x–044.x

Appendix 4. *International Classification of Diseases, 9th Revision, Clinical Modification* (ICD-9-CM) codes were used to identify the source of infection causing sepsis. Adapted from Esper et al. [3] and Wang et al. [4], and subsequently modified by us for HIV patients.

Organ System	ICD-9-CM Code	ICD-9-CM Code Description
Nervous		
	013	Tuberculosis of meninges and central nervous system
	036	Meningococcal infection
	091.81	Acute syphilitic meningitis (secondary)
	098.82	Neurosyphilis
	320	Bacterial meningitis
	321	Cryptococcal meningitis
	321.1	Meningitis in other fungal diseases
	324	Central nervous system abscess
	325	Phlebitis of intracranial sinus
	360	Purulent endophthalmitis
	376	Acute inflammation of orbit
	380.14	Malignant otitis externa
	383	Acute mastoiditis
Circulatory		
	093	Cardiovascular syphilis
	098.83 – 098.84	Gonococcal infections
	036.4	Meningococcal carditis
	391.2	Acute rheumatic myocarditis
	420.99	Acute pericarditis due to other specified organisms
	421	Acute or subacute endocarditis
Respiratory		
	010.1	Tuberculous pleurisy in primary progressive tuberculosis
	011	Pulmonary tuberculosis
	012	Other respiratory tuberculosis
	018	Miliary tuberculosis
	031.0	Pulmonary diseases due to other mycobacteria
	032	Diphtheria
	034	Streptococcal throat/scarlet fever
	098.6	Gonococcal infection of pharynx
	112.4	Candidiasis, of lung
	114.0	Primary coccidioidomycosis (pulmonary)
	114.2	Primary extrapulmonary coccidioidomycosis
	115.15	Histoplasma duboisii pneumonia
	115.05	Histoplasma capsulatum pneumonia
	115.95	Histoplasmosis pneumonia unspecified
	117.5	Cryptococcus neoformans
	117.3	Aspergillosis
	136.3	Pneumocystosis
	461	Acute sinusitis
	462	Acute pharyngitis
	463	Acute tonsillitis
	464	Acute laryngitis/tracheitis
	465	Acute upper respiratory infection of multiple sites/not

		otherwise specified
	475	Peritonsillar abscess
	480	Viral pneumonia
	481	Pneumococcal pneumonia
	482	Other bacterial pneumonia
	483	Pneumonia due to other specified organism
	485	Bronchopneumonia with organism not otherwise specified
	486	Pneumonia, organism not otherwise specified
	487.0	Influenza
	491.21	Acute exacerbation of obstructive chronic bronchitis
	494	Bronchiectasis
	510	Empyema
	513	Abscess of lung and mediastinum
Digestive		
	001	Cholera
	002	Typhoid/paratyphoid fever
	003	Other salmonella infection
	004	Shigellosis
	005	Other food poisoning
	008	Intestinal infections due to *Escherichia coli*
	008.1	Intestinal infections due to Arizona group of paracolon bacillus
	008.2	Intestinal infections due to *Aerobacter aerogenes*
	008.3	Intestinal infections due to *Proteus* (*Mirabilis morganii*)
	008.4	Intestinal infections due to unspecified bacteria
	008.5	Bacterial enteritis, unspecified
	009	Ill-defined intestinal infection
	014	Tuberculosis of intestines peritoneum and mesenteric glands
	129	Intestinal parasitism unspecified
	522.5	Periapical abscess without sinus
	522.7	Periapical abscess with sinus
	526.4	Inflammatory conditions of the jaw
	527.3	Abscess of the salivary glands
	528.3	Cellulitis and abscess of oral soft tissue
	540	Acute appendicitis
	541	Appendicitis not otherwise specified
	542	Other appendicitis
	562.01	Diverticulitis of the small intestine without hemorrhage
	562.03	Diverticulitis of the small intestine with hemorrhage
	562.11	Diverticulitis of colon without hemorrhage
	562.13	Diverticulitis of colon with hemorrhage
	566	Abscess of the anal and rectal regions
	567	Peritonitis
	569.5	Intestinal abscess
	569.61	Infection of colostomy or enterostomy
	569.83	Perforation of intestine
	572	Abscess of liver
	572.1	Portal pyemia
	575	Acute cholecystitis

Genitourinary		
	016	Tuberculosis of genitourinary system
	098.17	Gonococcal salpingitis specified as acute
	112.2	Candidiasis of other urogenital sites
	590	Kidney infection
	599	Urinary tract infection not otherwise specified
	601	Prostatic inflammation
	604	Orchitis and epididymitis
	614	Female pelvic inflammation disease
	615	Uterine inflammatory disease
	616.3	Abscess of Bartholin's gland
	616.4	Other abscess of vulva
Pregnancy		
	634	Spontaneous abortion, complicated by genital tract and pelvic infection
	635	Legally induced abortion, complicated by genital tract and pelvic infection
	636	Illegally induced abortion, complicated by genital tract and pelvic infection
	637	Unspecified abortion, complicated by genital tract and pelvic infection
	638	Failed attempted abortion, complicated by genital tract and pelvic infection
	639	Complications following abortion and ectopic and molar pregnancies
	646.6	Infections of genitourinary tract in pregnancy
	658.4	Infection of amniotic cavity
	670	Major puerperal infection
	675.1	Abscess of breast
Skin, soft tissue, or bone		
	003.24	Salmonella osteomyelitis
	015	Tuberculosis of bones and joints
	017	Tuberculosis of other organs
	031.1	Cutaneous diseases due to other mycobacteria
	035	Erysipelas
	036.82	Meningococcal arthropathy
	040.0	Gas gangrene
	095.5	Syphilis of bone
	098.5	Gonococcal infection of joint
	681	Cellulitis, finger/toe
	682	Other cellulitis or abscess
	683	Acute lymphadenitis
	685	Pilonidal cyst, with abscess
	686	Other local skin infection
	711	Pyogenic arthritis
	728.86	Necrotizing fasciitis
	730	Osteomyelitis
Other		
	790.7	Bacteremia
	958.3	Posttraumatic wound infection, not elsewhere classified

996.6	Infection or inflammation of device/graft
998.5	Postoperative infection
999.3	Infectious complication of medical care not otherwise classified

Appendix 5. *International Classification of Diseases, 9th Revision, Clinical Modification* (ICD-9-CM) codes for AIDS and CHC related diagnoses.

AIDS-related diagnoses	ICD-9-CM codes
Opportunistic infections	
Candidiasis (pulmonary or esophageal)	112.4, 112.84
Coccidioidomycosis, disseminated or extrapulmonary	114.1 – 114.4
Cryptococcosis (extrapulmonary)	117.5, 321.0
Cryptosporidiosis, chronic intestinal	007.4
Cytomegalovirus (other than liver, spleen, or nodes)	078.5
Herpes simplex: chronic ulcers or bronchitis, pneumonitis, or esophagitis	054.1 – 054.19, 054.71, 054.79
Histoplasmosis, disseminated or extrapulmonary	115.00 – 115.09 excluding 115.05, 115.10 – 115.19 excluding 115.15, 115.90 – 115.99 excluding 115.95
Isosporiasis, chronic intestinal	007.2
Mycobacterium avium complex or *Mycobacterium kansasii,* disseminated or extrapulmonary	031.2
Mycobacterium tuberculosis of any site	010.00 – 018.96
Mycobacterium, other species or unidentified species, disseminated or extrapulmonary	031.8, 031.9
Pneumocystis jirovecii pneumonia	136.3
Pneumonia (bacterial), recurrent	481.0 - 483.1, 484.3 – 484.5, and 484.8 - 486
Salmonella septicemia, recurrent	003.1
Toxoplasmosis of brain	130.0, 130.7, 130.9
Nervous system disease	
Encephalopathy	348.3, 348.30, 348.39
Progressive multifocal leukoencephalopathy	046.3
Cancer	
Cervical cancer, invasive	180.0 – 180.9
Kaposi sarcoma	176.0 – 176.9
Lymphoma, Burkitt	200.20 – 200.28
Lymphoma, immunoblastic	200.00 – 200.08
Lymphoma, primary, of brain	200.50 – 200.58
Wasting syndrome	
Wasting syndrome attributed to HIV	783.21, 783.22, 783.7, 260 – 263.9
CHC-related diagnoses	
Chronic liver disease and cirrhosis	
Chronic liver disease and cirrhosis	571.xx
Decompensated cirrhosis	
Awaiting organ transplant status	V49.83
Chronic HCV with hepatic coma	070.44
Unspecified hepatitis with hepatic coma	070.71

Encephalopathy not otherwise specified	348.3x
Esophageal varices in diseases classified elsewhere with or without bleeding	456.0, 456.1, 456.2x
Hepatic encephalopathy	572.2
Portal hypertension	572.3
Hepatorenal syndrome	572.4
Other sequelae of chronic liver disease	572.8
Jaundice	782.4
Ascites	789.5
Liver cancer	
Malignant neoplasm of liver and intrahepatic bile duct	155
Liver transplant	
Liver transplant	V42.7
Complications of transplanted liver	996.82
Liver transplant (auxiliary/other)	50.5x
Abuse of alcohol and drugs	
Alcohol dependence syndrome	303.xx
Drug dependence	304.xx
Nondependent abuse of drugs	305.xx
Drug Psychoses	292.xx
Alcoholic Psychoses	291.xx

Appendix 6. *International Classification of Diseases, 9th Revision, Clinical Modification* (ICD-9-CM) codes for comorbid diseases. Adapted from Cooke et al. [5].

Disease	ICD-9 CM Code Definition
Cardiovascular	390* to 429*, 440* to 459*
Infectious	001* to139*
Respiratory	460* to 519*
Gastrointestinal/Hepatic	520* to 579*
Neurologic	320* to 389*, 430* to 438*, 800* to 959*, E800 to E848*,
Trauma	E880* to E929*, E950 to E999*
Cancer	140* to 239*
Diabetes mellitus	250*

ICD-9-CM, International Classification of Diseases, Ninth Revision, Clinical Modification.
* represents inclusion of all fourth and/or fifth digit of the respective ICD-9 CM codes

REFERENCES

1. Angus DC, Linde-Zwirble WT, Lidicker J, Clermont G, Carcillo J, Pinsky MR. Epidemiology of severe sepsis in the United States: analysis of incidence, outcome, and associated costs of care. Critical care medicine 2001 Jul;29(7):1303-10.
2. Quan H, Sundararajan V, Halfon P, et al. Coding algorithms for defining comorbidities in ICD-9-CM and ICD-10 administrative data. Medical care 2005 Nov;43(11):1130-9.
3. Esper AM, Moss M, Lewis CA, Nisbet R, Mannino DM, Martin GS. The role of infection and comorbidity: Factors that influence disparities in sepsis. Critical care medicine 2006 Oct;34(10):2576-82.
4. Wang HE, Shapiro NI, Angus DC, Yealy DM. National estimates of severe sepsis in United States emergency departments. Critical care medicine 2007 Aug;35(8):1928-36.
5. Cooke CR, Hotchkin DL, Engelberg RA, Rubinson L, Curtis JR. Predictors of time to death after terminal withdrawal of mechanical ventilation in the ICU. Chest 2010 Aug;138(2):289-97.
